# SARS-CoV-2 Infection of Human Ovarian Cells: A Potential Negative Impact on Female Fertility

**DOI:** 10.3390/cells11091431

**Published:** 2022-04-23

**Authors:** Francesca P. Luongo, Filippo Dragoni, Adele Boccuto, Eugenio Paccagnini, Mariangela Gentile, Tamara Canosi, Giuseppe Morgante, Alice Luddi, Maurizio Zazzi, Ilaria Vicenti, Paola Piomboni

**Affiliations:** 1 Department of Molecular and Developmental Medicine, University of Siena, 53100 Siena, Italy; luongofrancescapaola@gmail.com (F.P.L.); tamara.canosi@student.unisi.it (T.C.); giuseppe.morgante@unisi.it (G.M.); paola.piomboni@unisi.it (P.P.); 2 Department of Medical Biotechnologies, University of Siena, 53100 Siena, Italy; dragonifilippo@gmail.com (F.D.); adele.boccuto@gmail.com (A.B.); ilariavicenti@gmail.com (I.V.); 3 Department of Life Sciences, University of Siena, 53100 Siena, Italy; eugenio.paccagnini@unisi.it (E.P.); mariangela.gentile@unisi.it (M.G.)

**Keywords:** SARS-CoV-2, granulosa cells, cumulus cells, female fertility

## Abstract

Severe acute respiratory syndrome coronavirus 2 (SARS-CoV-2) may affect female reproductive health. Here, we investigated the potential of SARS-CoV-2 to infect the follicular microenvironment, in particular granulosa (GCs) and cumulus cells (CCs), thus providing evidence for a productive infection. GCs and CCs were recovered from women (n = 25) who underwent in vitro fertilization at the Assisted Reproductive Unit, Siena University Hospital. Follicular ovarian cells were co-cultured with SARS-CoV-2 and then analyzed by qPCR, immunofluorescence (IF), western blot (WB) and transmission electron microscopy (TEM). In addition, cell culture supernatant was used to infect VERO6 cells. We demonstrated the expression of cell host factors ACE2, TRPMSS2, BSG and CTSL, which are pivotal for the virus life cycle. Cultured GCs and CCs incubated with SARS-CoV-2 revealed productive SARS-CoV-2 infection at 24 h, 48 h and 72 h post-adsorption. Indeed, SARS-CoV-2 RNA, spike and nucleocapsid proteins were detected in GCs and CCs, and their cell culture supernatant successfully infected the standard VERO E6 cells. Finally, TEM showed full-size virions attached to the membrane and located inside the cytoplasm. This in vitro study reveals the susceptibility of human ovarian cells to SARS-CoV-2 infection, suggesting a potential detrimental effect of COVID-19 infection on female human fertility.

## 1. Introduction

Coronavirus disease 2019 (COVID-19) is an acute respiratory infection caused by a novel coronavirus, SARS-CoV-2, first identified in Wuhan, China in December 2019. The incidence of COVID-19 first grew dramatically in China and then rapidly spread to more than 200 countries since late February 2020. On 11 March 2020, the World Health Organization declared the COVID-19 outbreak a global pandemic, and as of 25 November 2021, more than 250 million cases and 5 million deaths have been reported globally [1].

To enter host cells, SARS-CoV-2 uses the S1 subunit of the spike protein to bind the receptor angiotensin-converting enzyme 2 (ACE2) [2]. Then, the S2 spike subunit is cleaved at the S2 site by the host transmembrane serine protease 2 (TMPRSS2) or by the endolysosomal cathepsin L (CTSL) to produce unlocked, fusion-catalyzing viral forms. This priming step is necessary for viral entry [3,4,5]. In addition, basigin/CD147 (BSG) has been proposed as a potential additional host factor for SARS-CoV-2 [6], but this role has been not confirmed by formal analysis [7]. Following the fusion of viral and cellular membrane, viral RNA is released into the cytoplasm to generate multiple copies of the viral genome and direct the synthesis of structural and non-structural proteins in an ordered fashion. Finally, the N protein encapsulates viral RNA, budding into the endoplasmic reticulum–Golgi intermediate compartment, and the mature virion is then released from the cell surface where virus–host membrane fusion mediates viral dissemination [6].

Although COVID-19 has been characterized firstly by respiratory symptoms, strong evidence points to the infection of other tissues and organs [8]. Indeed, in humans, ACE2 expression has been detected in many organs, such as the respiratory tract, the gastrointestinal system, the kidney, heart, uterus, testis and placenta [9]. Noteworthy is that ACE2 is highly expressed in the human ovaries and in the stromal endometrial cells, as well as in granulosa cells (GCs) and oocytes in rat ovaries [9,10]. The expression of ACE2 is reported in the ovaries of reproductive-age and post-menopausal women [9], thus suggesting that the female reproductive system is potentially at risk of SARS-CoV-2 infection.

Female fertility is strictly dependent on the oocyte quality and competence. These oocyte characteristics are acquired within the ovarian follicle, where a bi-directional communication is established between the developing oocyte and the neighboring follicular somatic cells [11]. The latter include the GCs that line the follicle wall and the CCs that maintain tight contact with the developing oocyte. The mutual communication between the developing oocyte and somatic cells in the ovarian microenvironment is essential to develop full competence [12,13]. Indeed, the oocyte induces the proliferation of both GCs and CCs and, in turn, these cells are able to provide the oocyte with signals and molecules directly influencing its differentiation.

To the best of our knowledge, there are no studies demonstrating that somatic cells of the ovarian follicle can be infected by SARS-CoV-2. Here, we prove the ability of SARS-CoV-2 to infect the somatic ovarian cells with several assays, including real-time PCR, immunofluorescence, transmission and immunoelectron microscopy and the reinfection of reference SARS-CoV-2-susceptible cells by supernatants of virus-exposed GCs and CCs.

## 2. Materials and Methods

### 2.1. Study Design and Human Biological Sample Collection

The objective of this study was to assess whether human GCs and CCs may be infected by SARS-CoV-2. The cells used were from 25 women who underwent in vitro fertilization at the Assisted Reproductive Unit, Siena University Hospital. All the participants provided written informed consent for the use of their samples and data. The study was compliant with the Declaration of Helsinki and approved by the ethics committee of the University of Siena (CEAVSE, Protocol number 18370, 2 October 2020). 

### 2.2. Granulosa and Cumulus Cells Isolation

GCs were isolated from follicular fluid according to a previously described procedure [14]; CCs were collected after oocyte denuding, according to Valerio et al. [15]. The collected CCs and GCs were cultured and then washed twice, pelleted and stored for the following analyses.

### 2.3. SARS-CoV-2 Culture and In Vitro Infection

The SARS-CoV-2 strain, belonging to the lineage B.1 (EPI_ISL_2472896), was kindly provided by the Department of Biomedical and Clinical Sciences Luigi Sacco, University of Milan [16]. The VERO E6 cell line (ATCC^®^ CRL-1586) was maintained in High Glucose Dulbecco’s Modified Eagle’s Medium with sodium pyruvate and L-glutamine (DMEM; Euroclone) supplemented with 10% Fetal Bovine Serum (FBS; Euroclone) and 1% Penicillin/Streptomycin (Pen/Strep, Euroclone) in a humidified incubator at 37 °C with 5% CO_2_. CCs and GCs were cultured in DMEM supplemented with 10% FBS, 1% L-glutamine and 1% of Pen/Strep. The same propagation medium with a lower FBS concentration (1%) was used in the cell infection protocol. All procedures related to virus culture were carried out in a biosafety level 3 (BSL3) facility, according to WHO guidelines. Virus stocks were kept at −80 °C and titrated by plaque assay in VERO E6 cells, as previously described [17].

### 2.4. Infection of Granulosa and Cumulus Cells

CCs and GCs seeded in 6-well plates were infected with SARS-CoV-2 viral stock at the multiplicity of infection (MOI) 0.5. Following 1 h of adsorption at 37 °C with 5% CO_2_, the inoculum was removed, cells were washed once with PBS, and fresh medium was added (baseline or T0). Cells were cultivated for 3 days, and culture supernatants were collected at T0, at 24 h (T24) and at 48 h (T48) for virus titration by Tissue Culture Infectious Dose (TCID50) assay in VERO E6 and for quantification of viral RNA by Real-Time Quantitative PCR (qRT-PCR). CC and GC infection was tested in a single experiment, consisting of three cell cultures generated from three independent donors.

### 2.5. Culture Supernatant Titration by TCID50 Assay

For virus titration, VERO E6 cells were pre-seeded into 96-well plates to reach 70% confluency the day of infection. Log-5 dilutions of each CC and GC culture supernatant diluted in infection medium were used to infect VERO E6 cells in quadruplicate. After 1 h of adsorption at 37 °C with 5% CO_2_, the supernatant inoculum was removed, cells were washed with PBS, and fresh medium was added. Following 72 h incubation, the presence of the cytopathic effect (CPE) was observed by phase contrast microscopy, and cells’ viability was determined by quantifying the ATP produced by viable cells through the CellTiter-Glo 2.0 Assay (Promega), as previously published [18]. Luminescent signals were acquired with the GloMax^®^ Discover Microplate Reader (Promega). Cells were considered infected when the luminescent signal was twofold lower than the mean of the luminescent signal given by mock infected cells. A virus stock control was added to each plate, and each titration was performed in duplicate in VERO E6 cells. The virus titers were expressed as TCID50 per milliliter as determined by Reed & Muench’s analysis [19].

### 2.6. Detection of SARS-CoV-2 RNA

Viral RNA extraction was performed from CC and GC supernatant using the ZR Viral RNA kit (Zymo Research, Irvine, CA, USA), according to the manufacturer’s instructions. Then, cDNA was generated using ImProm-II™ Reverse Transcriptase (Promega, Madisson, WI, USA). Each cDNA was quantified in duplicate by qRT-PCR, with primers and probe designed on the NC_045512.2 reference strain targeting the SARS-CoV-2 polybasic cleavage site in the spike region. Conservation of primers and probes was checked on the consensus of the European circulating SARS-CoV-2 strains (GISAID, accessed in September 2020). To quantify viral RNA in culture supernatants, a standard curve obtained by diluting a plasmid rigorously quantified as described below was constructed in each qRT-PCR run and used to interpolate the cycle threshold (Ct) generated by supernatants.

To generate the plasmid used in the standard curve, a region of the spike gene spanning nucleotides 23,446–25,083 was inserted into the pGEM-T vector (Promega, Madisson, WI, USA) by TA-cloning. Following sequencing, serial dilutions of the plasmid were quantified by droplet digital PCR (ddPCR) using the QX200 Droplet Digital PCR System (Bio-Rad, Hercules, CA, USA). The reaction mixture included 5 μL of sample, 10 μL of ddPCR Supermix for probes no dUTP (Bio-Rad), 900 nM of each primer (P990–P991) and 250 nM of the FAM-labeled probe (P992). The plasmid quantified by ddPCR was used to generate the qRT-PCR standard curve, starting from 10 copies to 100,000 copies in 5 μL.

### 2.7. qRT-PCR

Total RNA was isolated from both GCs and CCs by using the automatic extractor Qiacube with the RNeasy Protect Mini kit (Qiagen, Hilden, Germany). Of the RNA, 100 ng were reverse-transcribed into cDNA using the iScript gDNA Clear cDNA Synthesis Kit (BioRad, Milan, Italy). Gene expression was evaluated using specific EvaGreen assays (Table S1), annealing only on the exon–exon junction sequence, ensuring selective amplification of the target genes, thus excluding genomic DNA contamination. To normalize the expression levels of the gene transcripts in CCs and GCs, a simultaneous mRNA expression profiling of the housekeeping genes *GAPDH* and *HPRT1* was included in all the analyzed samples. All amplification reactions were run in triplicate; melting curve analysis was also performed to confirm the specificity of the products obtained. Changes in gene expression levels were calculated by the 2^−ΔCt^ method.

### 2.8. Immunofluorescence

For immunofluorescence analysis, GCs and CCs were grown 24 h on coverslips, washed with PBS, and fixed for 15 min in 4% paraformaldheide (PFA). After blocking in 5% bovine serum albumin (BSA)/1% normal goat serum (NGS) in PBS for 30 min, cells were incubated overnight at 4 °C with the primary antibody diluted in 1% NGS, 1% BSA in PBS. After three washes with PBS, the cells were then incubated for 1hr at room temperature with the secondary antibody (Table S2). The nuclei were counterstained with DAPI in antifade solution. The samples were examined using a Leica 6500 microscope equipped with LAS software. Images were captured with a CFTR6500 digital camera (Leica, Wetzlar, Germany).

### 2.9. Western Blotting

Western blotting was carried out according to an in-house developed protocol [20] on pooled samples obtained by mixing equal amounts of total protein from five individual samples that were extracted in the same lysis buffer. After pooling, the protein concentration was measured again with the same quantification protocol. Of the total proteins from each pool, 50 μg were diluted in Laemmli buffer, kept at 95 °C for 5 min and separated on 10% polyacrylamide gel using the Cell Mini Protean (BioRad, Hercules, CA, USA, Microsciences, Wokingham, UK). After electrophoresis, the gel was transferred onto nitrocellulose membrane (Hybond ECL, Shrewsbury, UK, GE Healthcare, Chicago, IL, USA) in a Mini Trans-Blot apparatus (Bio-Rad, Hercules, CA, USA). The gel was then blocked for 1 h in 5% nonfat dry milk and then incubated overnight at 4 °C with primary antibodies (see Table S2) diluted in 1% nonfat dry milk/TTBS (TBS containing 0.2% Tween 20). After washing in TTBS, the membrane was incubated with the appropriate horseradish peroxidase (HRP)-conjugated secondary antibody (see Supplementary Table S2). The same nitrocellulose was also incubated with an anti–actin antibody (Biorad, Hercules, CA, USA, Microscience, Wokingham, UK), followed by the secondary antibody (Table S2), as an internal loading control. Immunostained bands were visualized by chemiluminescence with ImageQuant LAS 4000 (GE Healthcare, Chicago, IL, USA).

### 2.10. Transmission (TEM) and Immuno-Electron Microscopy (IMEM)

For TEM, SARS-CoV-2 infected cells were fixed in 2.5% glutaraldehyde diluted in 0.1 M, pH 7.2 cacodylate buffer (CB) for 1 h at 4 °C, washed in CB overnight, and then postfixed in 1% OsO_4_ for 1 h at 4 °C. Samples were dehydrated with a graded series of ethanol and embedded in epon resin. Ultrathin sections of 70 nm were collected on copper grids, stained with uranyl acetate and lead citrate, and observed with a FEI Tecnai G2 Spirit transmission electron microscope (Hillsboro, OR, USA).

For IMEM, infected cells were fixed in 4% paraformaldehyde, 0.1% glutaraldehyde in PBS for 1 h at 4 °C, washed in PBS, dehydrated with a graded series of ethanol, and embedded in LR White. Ultrathin sections of 100 nm were collected on nickel grids. The grids were incubated with 1% NGS, 1% BSA in PBS for 1 h at 4 °C, then overnight with primary antibody diluted in 1% NGS, 1% BSA in PBS at 4 °C, washed several times in 1% NGS, 1% BSA in PBS and again incubated with 10 nm gold-conjugated secondary antibody for 2 h at room temperature, washed in PBS and finally in distilled water. The grids were stained in uranyl acetate and in lead citrate before observation as above.

### 2.11. Statistical Analysis

Statistical analysis was performed using the GraphPad Prism 5.0 (GraphPad Software, San Diego, CA, USA), and statistical difference among groups of data was evaluated by the nonparametric Kruskal–Wallis test or the Mann–Whitney test. Statistical significance was set at *p* < 0.05.

## 3. Results

### 3.1. ACE2 Receptor and TMPRSS2, BSG, CTSL Accessory Proteases Are Expressed in Human Granulosa and Cumulus Cells

**Figure 1:**
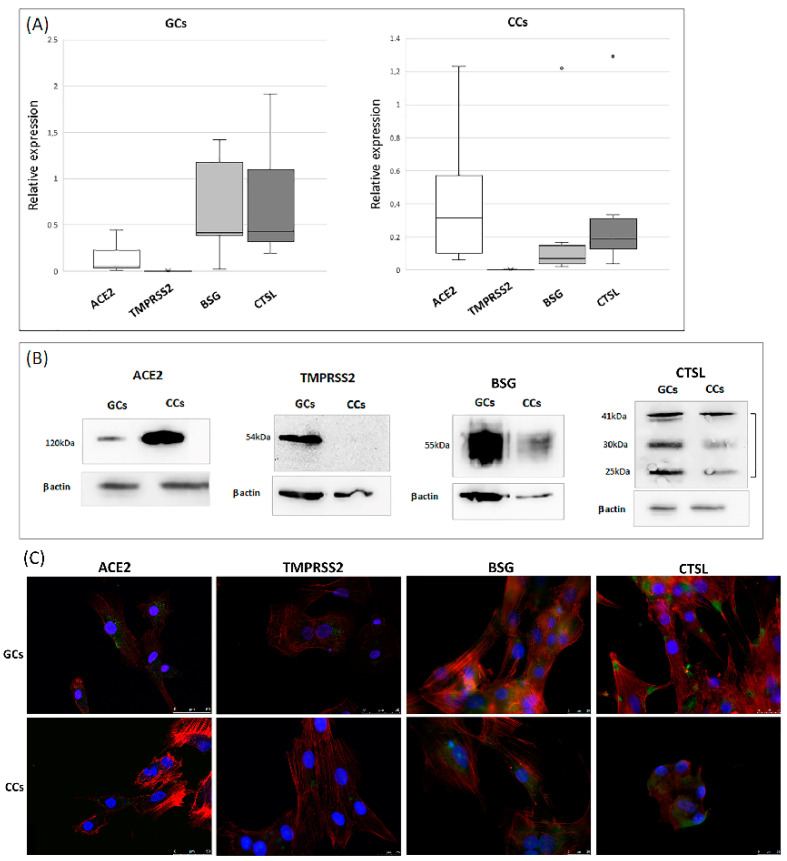
Expression and localization of ACE2 and its proteases in human GCs and CCs. (**A**) mRNA expression of ACE2, TMPRSS2, BSG and CTSL in human granulosa and cumulus cells. Graphical diagrams are plotted as box–whisker plots, where boxes show the interquartile range with median values, and whiskers represent min and max. Number of analyzed samples: GCs: 16, CCs: 16. (**B**) Western blot of ACE2, TMPRSS2, BSG and CTSL in human GCs and CCs. Western blots were repeated twice on a pool of 5 patients for each group, with comparable results. Equal protein loading of the two preparations was verified using the housekeeping beta-actin. (**C**) Representative micrographs (from three independent experiments) of immunofluorescence staining and confocal microscopy for SARS-CoV-2 host receptor ACE2 and TMPRSS2, BSG, CTSL proteases in human GCs and CCs. ACE2, TMPRSS2 and BSG are in green, actin is in red, nuclei are in blue. Scale bar = 50 μm.

The qRT-PCR analysis confirmed that ACE2 and TMPRSS2 were expressed in both GC and CC cells and highlighted the expression BSG and CTSL transcripts, even if at different levels. The ACE2 transcript was significantly increased in the CCs (0.43 vs. 0.15; *p* < 0.05), whereas BSG and CTSL were more expressed in GCs (0.7 vs. 0.3 and 0.8 vs. 0.4, respectively; *p* < 0.05) (Figure 1A). All genes were expressed in every tested sample, with the exception of TMPRSS2, which showed large inter-individual variability; in fact, we detected the expression of this gene in about 50% of CC samples (8 samples out of 16), whereas it was detectable in only 20% of the GCs (3 samples out of 16). Gene expression results were confirmed by western blot analysis. Indeed, ACE2 was most abundant in the cumulus cells, whereas CTSL were most expressed in granulosa cells, and the relative abundance of BSG was comparable in GCs and CCs (Figure 1B). Immunofluorescence cellular localization of ACE2, BSG and CTSL demonstrated an intense diffused signal (Figure 1C) on the plasma membrane in both GCs and CCs, whereas TMPRSS2 was detected as a weak staining on the membrane of these cells.

### 3.2. SARS-CoV-2 Infects Human Granulosa and Cumulus Cells

To prove the hypothesis that GCs and CCs are permissive to viral entry and replication, we infected in vitro primary human granulosa and cumulus cells with SARS-CoV-2.

**Figure 2:**
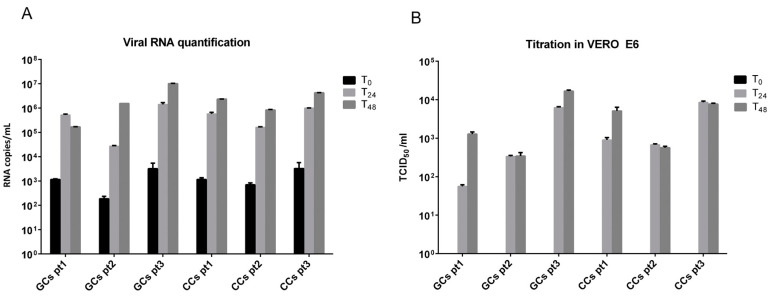
(**A**,**B**) SARS-CoV-2 replication in CCs and GCs cells is productive. (**A**) Viral RNA starts to be produced at 24 h and increases with respect to baseline (T0). (**B**) CC and GC supernatants collected at 24 h (T24) and 48 h (T48) are infective and quantifiable in VERO E6 cell lines.

Exposure of CCs and GCs to SARS-CoV-2 did not result in any visible cytopathic effect within the planned hours of observation. Consequently, to determine the permissibility of CCs and GCs to SARS-CoV-2 infection, supernatants were collected at different time points post-infection (T0, T24 and T48) and directly quantified by qRT-PCR or titrated through secondary infection of the reference VERO E6 cell line. Viral RNA titers in supernatants collected by CCs and GCs at T24 (Figure 2A) significantly increased (347 ± 140-fold) with respect to T0 in all patients (6.6 × 105 copies/mL at T24 vs 1.6 × 103 copies/mL at T0; *p* = 0.004). Viral RNA copies then remained stable at 48 h (3.2 × 106 copies per mL, mean for CCs and GCs in all patients). No significant differences were observed in the viral RNA amount between infected CCs and GCs. In Figure 2B are indicated the titers obtained, expressed as TCID50/ml. As expected, the virus collected after adsorption (T0) in CC and GC cells was not infectious; consequently, all titers obtained were not associated with a background signal but related to a real infection. Patient 3 showed the highest viral titer in CCs and GCs at each time point analyzed (3.9 ± 0.03 and 4.0 ± 0.3 log TCID50/mL for CCs and GCs, respectively, considering a mean between T24 and T48 collection); however, all cells were permissive for the infection and produce infectious virus, without significant differences with respect to the cell type. Twenty-four hours were sufficient to consistently infect the cells in all cases except one (patient 1, GCs 1.8 log TCID50/mL), which increased 22.6-fold at T48 collection (3.1 log TCID50/mL).

**Figure 3:**
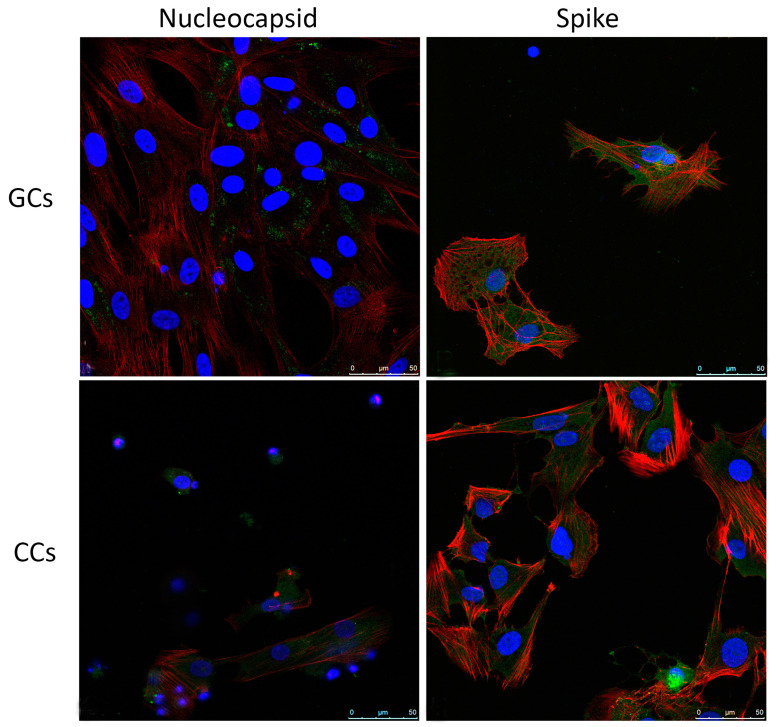
Intracellular localization of SARS-CoV-2 antigens. Representative images of immunofluorescence staining of Nucleocapsid and Spike n primary human GCs and CCs. Nucleocapsid and Spike (green), β-Actin (red). Nuclei were counterstained with 4,6-diamino-2-phenylindole (DAPI) (blue). Scale bar = 50 µm. GCs, n = 15; CCs, n = 15.

The viral infection of human GCs and CCs was additionally confirmed by immunofluorescence for spike and nucleocapsid virus proteins, evaluated at confocal microscopy (Figure 3). In both human GCs and CCs, nucleocapsid is distinguishable as dots in the cytoplasm, where spike protein is detected by a more diffuse staining.

### 3.3. Electron Microscopy Identification of SARS-CoV-2

**Figure 4:**
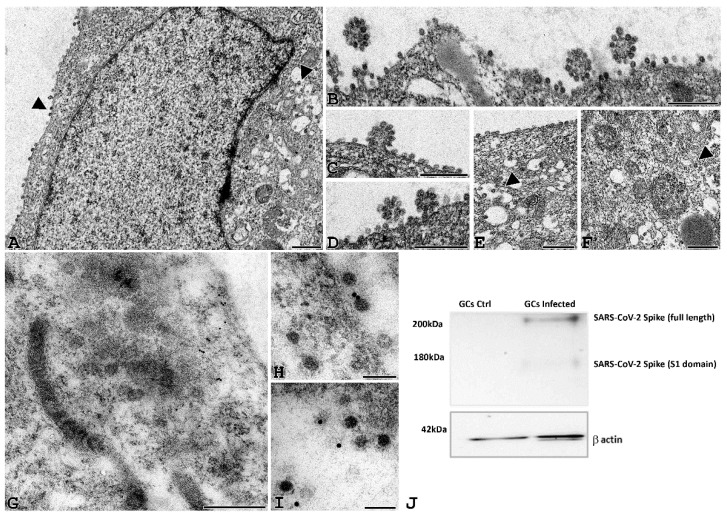
SARS-CoV-2 virions can be detected in GCs infected cells. (**A**–**F**) Representative electron microscopy images of GCs infected with SARS-CoV2 for 24 h. Viral particles are arranged around the cell membrane (**A**–**D**) and sometimes grouped in aggregates (**B**–**D**). Small viral infiltration clusters can be observed within the cytoplasmic compartment (**E**–**F**), and the ultrastructural organization is not altered (**A** bar = 1 µm; **B**–**F**, bar 500 nm). (**G**–**I**) Post-embedding immunogold electron microscopy localization of the spike protein. Localization of spike protein was shown in the cytoplasm of GCs (**G**, bar = 200 nm); in some cases, gold immunolocalization was observed close to viral particles (**H**,**I**, bar = 50 nm). (**J**) Western blot of SARS-CoV-2 spike in human GCs uninfected (GCs Ctrl) or infected with SARS-CoV-2 (GCs infected) for 24 h. The image shows a band of about 170 kD supporting the proteolytic activation of spike associated with viral entry. Equal protein loading of the two preparations was verified using the housekeeping beta-actin. Western blot was repeated twice on a pool of 3 patients.

TEM analysis of human GCs and CCs in vitro infected with SARS-CoV-2 was carried out at different time points post-infection (24 h, 48 h and 72 h) to analyze the time course of the viral binding and penetration. In Figure 4A–F, infected human GCs show cell-associated virus-like particles, similar to those visible among VERO E6 cells after incubation with SARS-CoV-2: virions were approximately spherical, with a diameter outside the lipid bilayer ranging from 50 to 150 nm and ultrastructural characteristics consistent with those described for other coronaviruses using cryo-EM [21]. Complete virions were also observed inside the cytoplasm as single or small groups of particles, either dispersed or within large vesicles. In addition, virus particles were identified while budding from the plasma membrane (Figure 4B–D).

To confirm these particles as SARS-CoV-2 virions, we performed an IMEM for spike protein in GCs infected for 24 h. As shown in Figure 4G–I, gold dots accounting for the spike protein were localized in the cytoplasm of granulosa cells, on viral particles. The presence of spike protein was definitively confirmed by western blotting analysis, also demonstrating proteolytic activation of this protein only in SARS-CoV-2-infected cells (Figure 4J).

**Figure 5:**
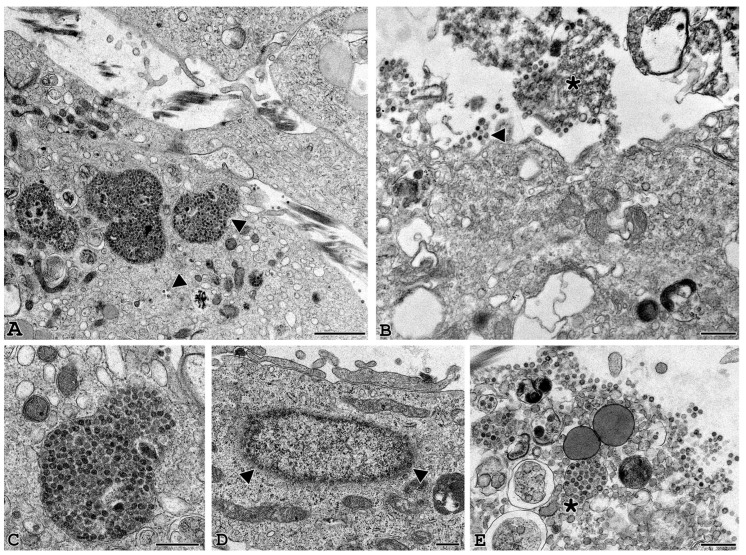
Longer exposure to SARS-CoV2 induces cell suffering and death. Representative electron microscopy images of granulosa cells at 48 h (**A**–**D**) and 72 h (**B**–**E**) post-infection. At 48 h, vesicles containing a large number of viral particles (**A**–**C**) are observed in the cellular compartment where the first signs of cellular suffering appear (asterisk). In addition, viral particles are seen near the nuclear membrane (**D**). At 72 h, cells appear ultrastructurally damaged (**B**–**E**) with clear signs of cell lysis and leakage of viral components. Arrowheads: viral particles. (**A**,**B**) bar = 1 µm. (**C**–**E**) bar = 500 nm.

Moreover, we investigated the ultrastructural features of GCs correlated with longer time virus exposure up to 48 and 72 **h** (Figure 5). At 48 h post-infection, the infection became massive, and the vesicles inside the cytoplasm contained a large number of viral particles near the nuclear membrane (Figure 5C,D). At this time, the first signs of cellular suffering appear and become more evident at 72 h, when the ultrastructural damage to the plasma membrane and cellular organelles, undergoing necrotic lysis as well as leakage of viral components, are clearly observed (Figure 5B–E).

## 4. Discussion

In this study, for the first time, we provide clear evidence of SARS-CoV-2 infection in granulosa and cumulus cells, the ovarian somatic cells that support oocyte development and competence acquisition. By detecting RNA, proteins and viral particles at ultrastructural level, we proved that these cells express the specific receptor ACE2 and the corresponding proteases TMPRSS2, BSG and CTSL, which allow the entry of this coronavirus into the host cell, by interacting in a coordinated manner with the viral spike protein.

The close relationship between oocytes and follicular cells raises the hypothesis that these cells may represent a vehicle for the oocyte SARS-CoV-2 infection, despite the hitherto unreported presence of this virus in somatic ovarian cells. Indeed, the possibility that the ovarian cells may be infected by SARS-CoV-2 has been recently reported in other studies, but no SARS-CoV-2 RNA, protein and particles were detected in these cells up to now. The presence of ACE2 and its accessory proteases in both GCs and CCs has been reported by indirect studies based on RNAseq databases and/or descriptive analyses of transcriptomic and proteomic data [22,23]. Moreover, the public databases Human Protein Atlas and Human Proteome Map report the presence of ACE2 protein in ovarian cells [22].

Here, we demonstrated that both GCs and CCs express ACE2 glycosylated full-length protein and its accessory proteases, TRPMSS2, BSG and CTSL, demonstrating the presence of an ideal environment for SARS-CoV-2 infection. We also provide evidence for their higher expression in CCs respectively to GCs. This datum is very interesting, since CCs are the follicular cells able to establish intimate connections with the developing oocyte (e.g., gap junctions). Therefore, contrary to what can be assumed, human cumulus cells cannot act as a barrier against virus entry into the oocyte. Rather, the ability of CCs to be infected by SARS-CoV2 may be a risk factor for the nearby oocyte. This is not obvious, as while some authors have reported the co-expression of ACE2 and its accessory proteases in the human oocyte [24]; others have shown that ACE2 is not expressed in the oolemma [25]. This may be related to an interindividual variability in the receptor of ACE2 expression, even if in our cohort of patients, we detected the presence in all the samples, analyzed by different approaches.

Two distinct routes for SARS-COV-2 entry, dependent on the target-cell proteases, have been reported [2]. After ACE2 binding, S protein activation may occur at the plasma membrane, where TMPRSS2 is expressed, whereas cathepsin-mediated activation occurs in the endosomal route, where TMPRSS2 is not present. Of note, some variants such as Omicron are capable of efficiently entering cells in a TMPRSS2-independent manner, via the endosomal route [26].

In our study, follicular cells, GCs and CCs have an interindividual variability and a very low expression of TMPRSS2, pointing to the fact that other proteases may have the main function, and TMPRSS2 is not solely necessary for the infection.

This is particularly true for CCs, where we were not able to detect any TMPRSS2 protein expression, but we proved the expression of both BSG and CTSL, confirming previously published data in both human and animal models [24,27,28]. Furthermore, the expression of CTSL in human GCs and CCs suggests the possible activation of membrane fusion within endosomes, thus confirming the ability of SARS-CoV-2 to establish robust infection through endosomal entry, as demonstrated in in vitro cell culture systems [29].

Moreover, transmission and immunoelectron microscopy showed that SARS-CoV-2 can enter and replicate in these cells, where the proteolytic activation of spike protein was also demonstrated. A recent study reported that conventional EM provides reliable information very similar to that obtained by cryo-EM, which at this time is the gold standard in structural biology [30]. By using EM, we demonstrated that SARS-CoV-2 particles in in vitro infected GCs are morphologically similar to those previously identified in the surrounding of the virus-producing VERO cells, with a diameter of the virions in the range of particle sizes reported by Lau et al. [30].

## 5. Conclusions

Being aware that our findings were obtained using an in vitro cellular model, we demonstrated that short-time exposures and low viral concentrations are able to cause infection. Cell damage seems to be directly related to the time of exposure; indeed, cell death is associated with prolonged exposure to SARS-CoV-2, supporting the high susceptibility of GCs to viral replication. This is a very important finding that offers an opportunity to critically revise the literature related to the effects of COVID19 on female fertility. Some studies report that there is no evidence that a history of asymptomatic or mild SARS-CoV-2 infection may negatively affect female fertility or ART outcomes, as well as the possibility of vertical transmission of SARS-CoV-2, in women with mild symptoms or those without symptoms of COVID–19 [31,32,33], except that the presence of S-protein on cumulus cells has be highlighted [34,35].

This study provides the first biological explanation for the recently reported evidence that infection with SARS-CoV-2 could impair ovarian function, thus potentially affecting reproductive outcomes [36].

## Data Availability

All data underlying the study are available from the corresponding authors.

## Supplementary Materials

The following supporting information can be downloaded at https://www.mdpi.com/article/10.3390/cells11091431/s1: Table S1: ID and sequences of primer used to detect gene expression; Table S2: List of antibodies used in this study.
