# Supplementary material for: SARS-CoV-2 Infection of Human Ovarian Cells: A Potential Negative Impact on Female Fertility
Source: Cells. 2022 Apr 23;11(9):1431. doi: 10.3390/cells11091431 (PMC9105548; doi:10.3390/cells11091431)
Supplement: Supplementary file 1 [file cells-11-01431-s001.zip › cells-1658213-supplementary.pdf]

Supplementary Materials

Table S1. ID and sequences of primer used to detect gene expression.

Target Genes	Acronym	ID assay/primer sequence
Angiotensin-converting enzyme 2	ACE2	Hs.PT.58.27645939
Transmembrane serine protease 2	TMPRSS2	Hs.PT.58.39408998
Cathepsin L	CTSL	Hs.PT.58.45751460
Basigin	BSG	Hs.PT.56a.39293590.g
Spike	SPIKE	5′GGCACGTAGTGTAGCTAGTC3′<br>5′TGGGTATGGCAATAGAGTTATTA G3<br>5′FAM-ACTATGTCACTTGGT-MGB3′
**Reference Genes**	**Acronym**	**ID assay**
HypoxanthineGuanine PhosphoribosylTransferase 1	HPRT1	Hs.PT.58v.45621572
Glyceraldehyde 3-phosphate dehydrogenase	GAPDH	Hs.PT.58.589810.g

Table S2. List of antibodies used in this study.

Antigen	Donor species	Dilution		Manufacturer	RRID
		IF	WB		
Primary antibodies					
β-Actin	Mouse	-	1:3000	Bio-Rad Laboratories	AB_2223350
ACE-2	Mouse	1:250	1:1000	R&D System	AB_355722
TMPRSS2	Mouse	1:200	1:10	Santa Cruz Technology	AB_2205599
CTSL	Mouse	1:500	1:100	Santa Cruz Technology	AB_626811
BSG	Mouse	1:500	1:100	Santa Cruz Technology	AB_626911
SARS-CoV2 Nucleocapsid	Mouse	1:1000	1:500	Bio-techne	AB_1522790
SARS-CoV2 Spike	Rabbit	1:1000	1:1000	Bio-techne	AB_1237374
Secondary antibodies					
Anti-Mouse IgM FITC	Goat	1:500	-	Sigma Life Science	AB_259799
Anti-Rabbit IgM FITC	Goat	1:200	-	Sigma Life Science	AB_1137637
Anti-Mouse-IgG HRP	Goat	-	1:5000	Bio-Rad Laboratories	AB_609692
Anti-Rabbit-IgG HRP	Goat	-	1:5000	Bio-Rad Laboratories	AB_1102634
